# Gunshot Wound of the Crural Artery and Vein, above the Profunda Artery and Vein

**Published:** 1881-01

**Authors:** P. Kraske, Herman Mynter


### Translations.

### GUNSHOT WOUND OF THE CRURAL ARTERY AND VEIN, ABOVE THE PROFUNDA ARTERY AND VEIN—LIGATURE OF THE VESSELS—GANGRENE—DEATH. FROM VOLKMAN'S CLINIC, BY DR. P. KRASKE.

FROM THE GERMAN BY HERMAN MYNTER, M. D.

A HEALTHY and vigorous student, 21 years of age, was wounded in a duel (distance 15 steps) July 5, 1880, with a bullet of smallest calibre. The bullet entered on the anterior and outer side of the right femur, just below the inguinal sulcus, and a gush of arterial blood showed that the large vessels were injured. There was no point of exit. The bleeding was stopped by means of Esmarck's bandage, and the wounded man carried to the clinic. He was then put under the influence of chloroform, and while the vessels were compressed above and below, the wound was dilated and the vessels exposed. The artery, lying in a large extravasate of blood, was found transversely perforated just below Poupart's ligament, and was ligated double. The compression being omitted a new gush of dark-colored blood occurred, showing that the vein, too, was wounded, but the wound could not be discovered, as the blood gushed out as soon as the compression was stopped. The vein was therefore tied below the suspected point, and as the bleeding continued from above, it was likewise ligated higher. As the bleeding still did not cease, the vein was ligated above Poupart's ligament. The vessels were exposed for a distance of 15 centimeters before the bleeding ceased, and the vein was found laterally wounded a little above the wound in the artery. The bullet could not be found and was supposed to have buried itself in the loose cellular tissue between the blood and the symphysis.

Before the ligatures were applied the leg felt cold and the patient was found unable to move it about. As many subcutaneous veins were also injured, it was evident that gangrene would follow. Yet, exarticulation in the hip-joint was not performed, as the patient, on account of the loss of blood during the operation, which had lasted two hours, was in such a state of collapse that he would have died on the table. The wound (30 centimeters long) was disinfected, a drainage tube inserted, and Lister's antiseptic bandage applied. No sutures were used.

The chloroform narcosis being over, there was no feeling whatever in the leg, but the next morning (July 6th) the skin felt warm to the knee, looked reddish and the prick of needles could be felt. Below the knee dilatation of the veins, cold and insensibility was noticed. Temperature toward evening 102½.

July 7. Evening temperature 104; his condition bad, with dry tongue and burning thirst, no swelling of the wound, and but slight odor; circulation seems restored to the knee, and on the flexor side a little farther downwards, gangrene of the calf commenced. The muscles in the wound show a grayish-brown discoloration throughout their whole thickness.

July 9. It is more and more evident, that while the circulation is restored in the skin of the thigh, the muscles are necrotic up to the wound. In the wound they have become dry and firm, and it looks as if munification had occurred. There is no redness or swelling of the wound, which looks just as it did the first day.

July 11. The patient died in collapse.

At the legal post-mortem examination a large bloody infiltration was found in the surroundings of the wound and in the subperitoneal tissue up to the right kidney. In the ligated artery and vein firm thrombi were found. The gangrene of the muscles reached much higher up than in the skin, in the muscles on the anterior and inner side of the femur to the wound, on the external and posterior side to the middle of the femur.

The author, after reporting this case, discusses some points of interest as follows:

The wound and ligature of the femoral artery and vein occurred in this case in a spot which is of great surgical interest. On account of the distribution of the valves in the lateral branches, the femoral vein at Poupart's ligament is, according to Braune, the only venous trunk, through which the blood may pass from the lower extremity into the abdominal cavity, and a ligature on this particular spot must, therefore, he thinks, necessarily be followed by gangrene.

We might take the case reported as a proof of the correctness of the assertions of Braune, but, in this case both artery and vein were ligated and it is therefore necessary first to study, how the ligature of the vein was influenced by the ligature of the artery. This is the more necessary from the fact that very recently the opinion has prevailed, that the simultaneous ligature of a large artery and vein must necessarily be followed by gangrene and that, therefore, immediate amputation was indicated. Langenbeck claims that this opinion is not only erroneous, but that, on the contrary, the ligature of a large vein is favorably influenced by simultaneous ligature of the corresponding artery. While he and other surgeons have observed severe disturbances in the circulation, after ligature of the jugular vein, no symptoms occurred in two cases, in which he simultaneously ligated the common jugular vein and the common carotic artery; similar observations have been made on other large veins. Volkman, for instance, extirpated a myxosarcoma as large as the head of a child, from the left femur, and was obliged to remove about 4 inches of the femoral arteries and vein, just below the arteria profunda. No disturbances of the circulation occurred.

Rose ligated simultaneously the femoral artery and vein on account of a stabbed wound, and the patient recovered. Pettingen ligated the crural vein during the extirpation of a tumor. As the bleeding continued and the leg assumed a cyanotic color, he ligated the arteries also, and then the cyanosis disappeared. Similar observations have been made by experiments upon animals. If in the case of a rabbit we ligate the large veins of the leg on one side, and on the other leg we simultaneously ligate the artery and the vein, no disturbances of the circulation will occur in the latter, while in the former œdematous swelling around the tendon Achilles will set in. The differences in the circulation under similar circumstances may be studied with the microscope on the webbed foot of a frog. On the side on which the vein alone is ligated, we see the picture of stasis of the blood, as Cohnheim has described it. All veins and capillaries are greatly dilated and filled with blood-corpuscles, which move very slowly or not at all. On the other leg, of which both artery and vein are ligated, we see, to be sure, numerous capillaries shut off from the circulation, and seen either empty or containing blood corpuscles, which do not move, but nowhere are the vessels so dilated or so excessively filled with blood corpuscles as on the other leg; and where the blood circulates at all it circulates in a constant stream.

We may therefore consider it as proved, that the disturbances in the circulation, following the ligature of a venous trunk are generally favorably influenced by the simultaneous ligature of the corresponding artery. I say generally, because it is evident, that if there are not sufficient collateral branches present above the point of ligature, if, in short, the ligated artery is a terminal branch, gangrene is an inevitable result. This was not the case in the example reported. Arterial collateral branches were present in sufficient numbers, as after the ligature of the artery the flow continued from the peripheral end of the vein, and the ligature of the vein must have been the cause of the primary gangrene which occurred. The case, therefore, proves the correctness of the ideas of Braune.

If we examine the published cases of ligature of the femoral vein at Poupart's ligament, we find that in all cases in which the vein was ligated on account of traumatic injuries, gangrene followed; while in the cases in which the vein was ligated on account of extirpation of tumors generally no gangrene occurred. The supposition that collateral branches had been developed gradually during the increasing pressure of the growing tumor, is so much more probable, as in the cases in which gangrene followed the extirpation of tumors, they were growing rapidly. Possibly individual peculiarities in the distribution of the vessels, or insufficiency of the valves may be taken into consideration, but, generally speaking, it is a fact that a sudden closure of the femoral vein at Poupart's ligament cuts off the circulation totally in the leg, while by a gradual closure dilatation and formation of collateral branches is possible. The practical point is, therefore, that the femoral vein may be ligated in cases of extirpation of slowly growing tumors. In cases of traumatic injuries of the normal femoral vein above the vasa profunda and the vena saphena magna ligature of the vein is extremely dangerous. In these cases it is very difficult to stop the bleeding. Langenbeck's advice, to ligate the artery, does not always attain the object, as Stromeyer, Bilroth and Rose have shown. Lateral ligature and tampon are unsafe. If used at all, we ought to bandage the leg firmly and place it in an extended and outwardly rotated position, as, according to Braune, the femoral vein in this position is bloodless and collapsed. In bad cases the advice of Rany and Lindhart, to immediately exarticulate at the hip-joint, ought to be followed.

The author calls attention to one more point of interest, the different extension of the gangrene in the skin and in the muscles. The skin sloughed only below the knee, while the gangrene in the muscles extended up toward the wound. The cause of this phenomena is, that there does not exist sufficient anastomoses between the skin and the muscles. The muscles have, as has been shown in the laboratory of Ludwig, an independent circulation, which has no direct communication with that of the neighboring organs.

This fact is not without practical interest and the author mentions two cases, of Lücke and Mack, in which, after traumatic injury, in one case of tibialis anticus, and extensor digitorum communis, in the other of gluteus maximus, gangrene of muscles occurred, while the skin remained intact. A similar case of traumatic necrosis of the muscles of the thigh is mentioned by Volkman. Amputation became necessary, following which there occurred almost complete sloughing off of all the muscles.—(*Centralblatt fuer Chirurgie*, No. 43, 1880.)

